# TLR7/9 antagonist reduces HIV-1-induced immune activation

**DOI:** 10.1186/1742-4690-9-S2-P172

**Published:** 2012-09-13

**Authors:** J Chang, RJ Lindsay, EH Doyle, V Vrbanac, EN Seung, T Dudek, RJ Bosch, M Precopio, E Kandimalla, A Tager, M Altfeld

**Affiliations:** 1 Ragon Institute of MGH, MIT and Harvard, Boston, MA, USA; 2 Massachusetts General Hospital, Boston, MA, USA; 3 Harvard School of Public Health, Boston, MA, USA; 4 Idera Pharmaceuticals Inc., Cambridge, MA, USA

## Background

T cell immune activation is a strong predictor of HIV-1 disease progression and HIV-1 transmission, and IFN-alpha production following TLR7 stimulation has been associated with elevated CD8^+^ T cell activation (Meier et al., Nat Med 2009). We therefore hypothesized that modulation of TLR7 stimulation could be used to manipulate IFN-alpha production and subsequently reduce HIV-1-associated immune activation. TLR7/9-specific antagonists developed for treatment of auto-immune diseases were used in vitro and in vivo in a humanized mouse model.

## Methods

Humanized BLT mice were generated by transplanting irradiated NOD/SCID/γc^-/-^ mice with human fetal thymus and injected with human hematopoietic stem cells isolated from matching liver tissue. Following reconstitution, cells were harvested from the mice to examine the effects of the antagonist in vitro. Humanized mice were also infected with HIV-1 and then either treated or untreated with TLR7/9 antagonist from Idera. T cell activation markers were examined pre-infection, following infection and during treatment with the antagonist. Additionally, responsiveness of DCs to TLR7/8 stimulation ex vivo following in vivo TLR7/9 antagonist treatment was assessed by intracellular cytokine staining.

## Results

16-20 weeks after transplant, human DC, monocyte and T cell populations were detectable in the mice. Ex vivo stimulation with TLR7/8 induced cytokine production by humanized mice DCs and monocytes similar to those observed from human PBMCs and this was significantly blocked by in vitro treatment with the TLR7/9 antagonist (P<0.05). HIV-1 infection of humanized mice led to increased T cell immune activation marker CD38 on human T cells in vivo, and treatment of infected mice with the TLR7/9 antagonist led to a significant reduction in CD38 expression.

## Conclusion

Treatment of HIV-1-infected humanized BLT mice with a TLR7/9 antagonist resulted in a significant reduction of HIV-1-associated immune activation. This may have important implications in reducing viral transmission associated with higher immune activation in HIV-1.

